# Enhancing Post-Stroke Rehabilitation and Preventing Exo-Focal Dopaminergic Degeneration in Rats—A Role for Substance P

**DOI:** 10.3390/ijms23073848

**Published:** 2022-03-31

**Authors:** Sibylle Frase, Franziska Löffler, Jonas A. Hosp

**Affiliations:** Department of Neurology and Neuroscience, Medical Center—University of Freiburg, Faculty of Medicine, University of Freiburg, 79106 Freiburg, Germany; sibylle.frase@uniklinik-freiburg.de (S.F.); franzi.loeffler@web.de (F.L.)

**Keywords:** photothrombotic stroke, exo-focal neurodegeneration, motor rehabilitation, substance P

## Abstract

Dopaminergic signaling is a prerequisite for motor learning. Delayed degeneration of dopaminergic neurons after stroke is linked to motor learning deficits impairing motor rehabilitation. This study investigates safety and efficacy of substance P (SP) treatment on post-stroke rehabilitation, as this neuropeptide combines neuroprotective and plasticity-promoting properties. Male Sprague Dawley rats received a photothrombotic stroke within the primary motor cortex (M1) after which a previously acquired skilled reaching task was rehabilitated. Rats were treated with intraperitoneal saline (control group, *n* = 7) or SP-injections (250 µg/kg) 30 min before (SP-pre; *n* = 7) or 16 h (SP-post; *n* = 6) after rehabilitation training. Dopaminergic neurodegeneration, microglial activation and substance P-immunoreactivity (IR) were analyzed immunohistochemically. Systemic SP significantly facilitated motor rehabilitation. This effect was more pronounced in SP-pre compared to SP-post animals. SP prevented dopaminergic cell loss after stroke, particularly in the SP-pre condition. Despite its proinflammatory propensity, SP administration did not increase stroke volumes, post-stroke deficits or activation of microglia in the midbrain. Finally, SP administration prevented ipsilesional hypertrophy of striatal SPergic innervation, particularly in the SP-post condition. Mechanistically, SP-pre likely involved plasticity-promoting effects in the early phase of rehabilitation, whereas preservation of dopaminergic signaling may have ameliorated rehabilitative success in both SP groups during later stages of training. Our results demonstrate the facilitating effect of SP treatment on motor rehabilitation after stroke, especially if administered prior to training. SP furthermore prevented delayed dopaminergic degeneration and preserved physiological endogenous SPergic innervation.

## 1. Introduction

Ischemic stroke is characterized by a necrosis of brain tissue directly caused by an occlusion or hypo-perfusion of a supplying arterial vessel [1]. In addition, selective loss of neuronal populations may occur far away from the ischemic lesion with a delay of several days to a few weeks [2,3]. This delayed and exo-focal neurodegeneration after stroke is particularly well investigated in ipsilesional dopaminergic midbrain nuclei: here, neurodegeneration has been described in rats [2,4], mice [5,6] and even humans [7,8]. Although secondary neurodegeneration is commonly investigated in the model of middle cerebral artery occlusion (MCAO) [2,4,5,6], we have recently reported an unexpected widespread secondary loss of dopaminergic midbrain neurons in a model of photothrombotic stroke (PTS) within the primary motor cortex (M1) of rats [9].

Secondary dopaminergic degeneration after stroke has been linked with unfavorable outcomes such as post-stroke depression [5] or parkinsonism [10]. A dopaminergic deficiency in stroke survivors furthermore impairs elementary motor encoding [11] and motor skill acquisition [12]. These observations are not surprising, as dopaminergic signaling is crucially involved in motor-learning dependent plasticity in M1 (for review see [13]): dopamine induces learning-related transcription factors, mediates synaptic long-term plasticity, increases cortical excitability and improves sensory-motor feedback. Consequently, key molecules of dopaminergic signaling (e.g., dopamine receptors) become up-regulated within M1 in response to motor training [14]. Dopaminergic neurons projecting to M1 are located within the ipsilateral ventral tegmental area (VTA), whereas the integrity of this projection is a prerequisite for successful motor learning [15]. Interestingly, loss of dopaminergic neurons within the VTA has been also observed after a photothrombotic stroke within M1 [9].

As motor learning is considered to be an essential mechanism for recovery of motor deficits post stroke [16], the treatment of dopaminergic deficiency has been identified as a therapeutic approach to support motor rehabilitation [17]. In Humans, levodopa-substitution yielded mixed results [18,19,20] as it is complicated by the necessity of strict administration intervals [20] and the problem of proper dosing due to genetic polymorphisms of molecules involved in dopamine signaling [21]. To overcome the necessity of levodopa substitution, the concept of preventing post-stroke dopaminergic degeneration using neuroprotective agents such as the anti-excitotoxic drug MK-801 [6] or the antidepressant citalopram [5] has been successfully applied in mice. In this context, the application of substance P (SP), an eleven amino acid peptide of the tachykinin family [22] would be a further promising approach, as it may treat dopaminergic deficiency after stroke via multiple mechanisms: SP exerts a protective effect against excitotoxic damage [23] and deprivation of trophic factors [24]. This protective effect is particularly well documented for dopaminergic neurons [25,26]. Furthermore, SP is considered a “mnemonic” molecule [27] as its application supported hippocampus [28]- and amygdala-based learning paradigms [27]. Within M1, injecting SP facilitated the acquisition of a skilled reaching task in rats [29]. Thus, besides neuroprotection, SP could also support neurorehabilitation by promoting neuroplasticity. In addition, there is evidence for an involvement of the SPergic system within the post-stroke degeneration of dopaminergic neurons: SP synthesizing striatal neurons densely innervate dopaminergic midbrain nuclei [30]. After experimental strokes involving the striatum, a loss in nigral SP has been observed as a function of striato-nigral differentiation [31,32]. However, after a pure cortical stroke with spared striato-nigral connections, an opposite nigral expression pattern with increased SPergic innervation of midbrain nuclei emerged [9], potentially indicating a compensatory mechanism. 

Here, we tested the effect of systemic SP administration on motor rehabilitation after a photothrombotic stroke (PTS) targeting M1 in rats. The protective propensity of SP on dopaminergic neurodegeneration was assessed histologically. To further segregate a pure protective from additional pro-neuroplastic effects, SP was administered at two timepoints: 30 min before and 16 h after rehabilitation training. As SP is known to be a pro-inflammatory agent [33], we furthermore histologically assessed microglial activation to determine the inflammatory response within the tegmental midbrain.

## 2. Results

### 2.1. Intraperitoneal Substance P Application Facilitates Motor Rehabilitation after Stroke

**Figure 1:**
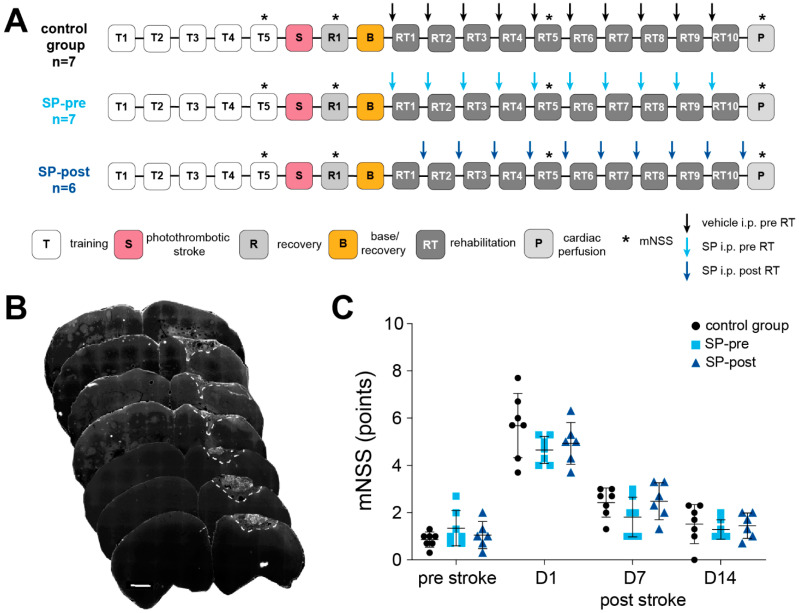
(**A**) Schematic depiction of the experimental timeline. (**B**) Series of microscopy images (10× magnification) displaying a representative photothrombotic ischemic stroke affecting the primary motor cortex (M1), identified by Fluorojade-C staining. (**C**) A similar stroke-related neurological deficit measured by the modified neurological severity score (mNSS) that recovered with time was present in all groups. Scale bar: 500 µm. Data are presented as mean ± SEM.

**Figure 2:**
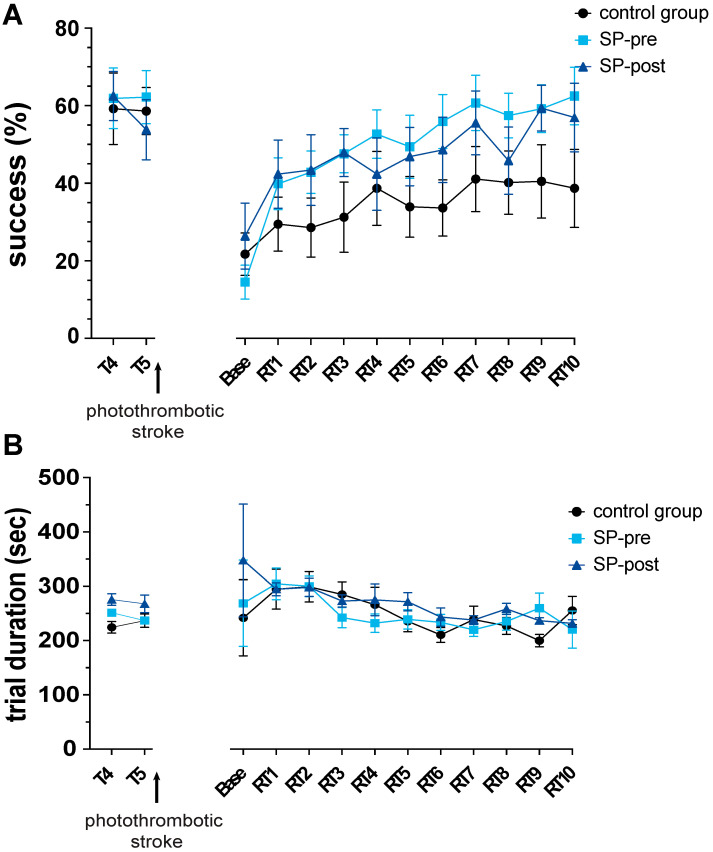
Intraperitoneal Substance P application facilitates motor rehabilitation after stroke. (**A**) After stroke, all groups showed a similar drop in performance. Administration of SP (SP-pre: 250 μL i.p. 30 min before training sessions RT1-10; SP-post 250 μL i.p. 16 h after training sessions RT1-10) significantly improved rehabilitation success compared to saline-treated controls. (**B**) Trial durations as a measure of motivation did not differ between groups. Data are presented as mean ± SEM.

There was no significant difference in stroke volumes between groups (CG: 0.696 ± 0.36 mm^3^, *n* = 6; SP-pre: 0.57 ± 0.38 mm^3^, *n* = 6; SP-post: 1.48 ± 1.3 mm^3^, *n* = 6; 1-way ANOVA t(df) = 2.91(2, 15); *p* = 0.15). Body weight was not different between groups throughout the experiment (mixed-effects analysis F(2, 16) = 0.12, *p* = 0.89). As indicated by the mNSS, a stroke-related neurological deficit which recovered with time was present in all animals (Figure 1C; RM-ANOVA mean effect of time: F(2.24, 35.80) = 138.9, *p* < 0.0001). However, there was no significant difference between groups (F(2, 16) = 1.19, *p* = 0.33) or with respect to group x time interaction (F(6, 48) = 1.56, *p* = 0.18). There was no difference in plateau performance in the reaching task (days 4 and 5 of pre-stroke training) between groups (success rate CG: 60.72 ± 20.48%, *n* = 7, SP-pre: 61.91 ± 18.44%, *n* = 7, SP-post: 58.16 ± 16.98%, *n* = 6; 1-way ANOVA F(2, 17) = 0.066 *p* = 0.94). All groups showed a similar drop in reaching performance post stroke, indicating comparable stroke-induced motor deficits (Figure 2A; CG: 38.99 ± 18.81%, SP-pre: 47.38 ± 8.83%, SP-post: 31.77 ± 22.72%, F(2, 17) = 1.30, *p* = 0.30). 

Regarding rehabilitation training (Figure 2A), the factors time (RM-ANOVA mean effect of time: F(3.66, 58.96) = 12.63, *p* < 0.01) and group (F(2, 16) = 4.19, *p* = 0.034) significantly influenced recovery of function, the interaction effect time x group was different at trend-level (F(7.37, 58.96) = 1.89, *p* = 0.084). Regarding rehabilitation plateaus, SP-pre animals showed the best recovery, followed by animals of the SP-post group (control group 18.06 ± 18.26%, SP-pre 45.20 ± 17.25%, SP-post 27.66 ± 15.89%). Regarding trial durations that were assessed as a measure of motivation (Figure 2B), a non-significant trend was present for an acceleration over time (RM-ANOVA effect of time: F(1.711, 28.58) = 3.11, *p* = 0.067), whereas group had no effect (F(2, 17) = 0.39, *p* = 0.68). 

### 2.2. Dopaminergic Neurodegeneration after Stroke Is Attenuated by Substance P 

**Figure 3:**
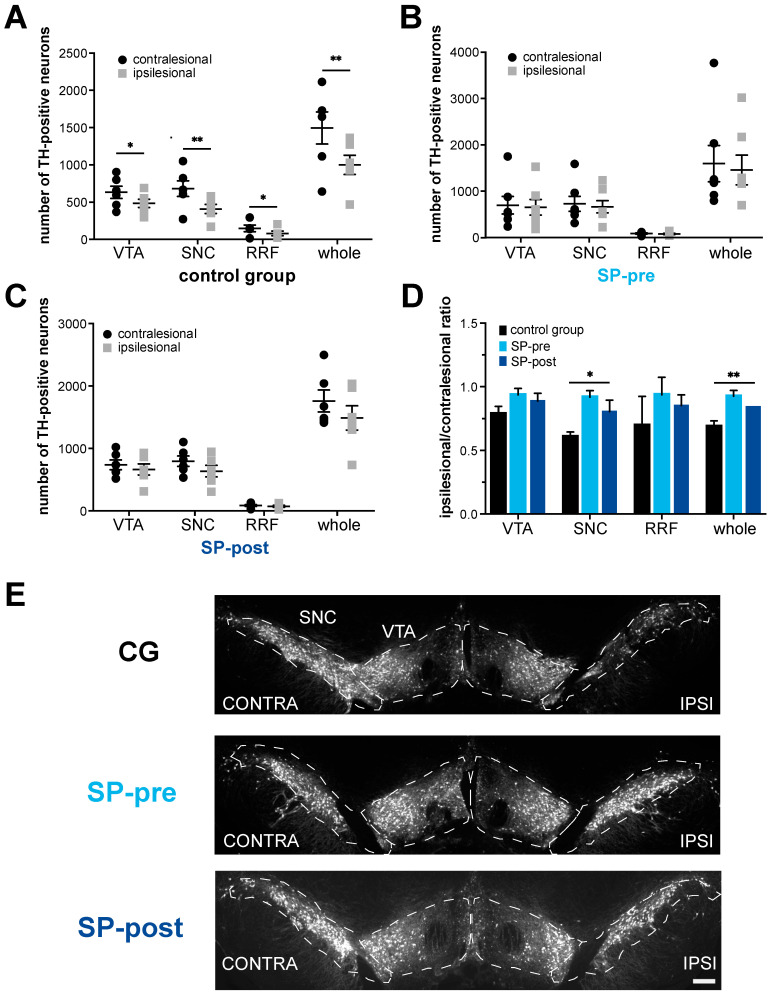
Dopaminergic neurodegeneration after stroke is attenuated by Substance P. (**A**) Intraindividual comparison of TH-positive neuron counts between contra- and ipsilesional hemisphere revealed ipsilesional neuron loss in control animals. (**B**,**C**) In SP-pre and SP-post animals, no ipsilesional dopaminergic degeneration was observed. (**D**) Between-group comparisons of ipsilesional/contralesional TH-positive cell count ratios revealed significant differences between groups for the whole hemisphere and SNC. (**E**) Representative light-microscopy images (10× magnification) indicate bilateral midbrain TH-immunoreactivity 15 days after stroke induction. Please note the obvious loss of TH-positive neurons within the ipsilesional SNC of a control rat. Scale bar: 100 µm. RRF: retrorubral field, SNC: substantia nigra pars compacta, VTA: ventral tegmental area. * *p* < 0.05, ** *p* < 0.01. Data are presented as mean ± SEM.

After stroke, TH-positive neurons were reduced by 33% in the ipsilesional compared to the contralesional midbrain in the control group (Figure 3A, ipsi: 1002 ± 315.7 cells, contra: 1496 ± 526.4 cells, *n* = 6, paired *t*-test *p* = 0.0079). Considering mesencephalic subfields, TH-positive neurons were reduced significantly in the ventral tegmental area (ipsi: 484.8 ± 135 cells, contra: 632.2 ± 200.8 cells, paired *t*-test *p* = 0.028), the substantia nigra pars compacta (ipsi: 409.5 ± 149.0 cells, contra: 681.5 ± 256.6 cells, paired *t*-test *p* = 0.0046, Figure 3E) and the retro rubral field (ipsi: 81.0 ± 71.84 cells, contra: 148.2 ± 99.01 cells, paired *t*-test *p* = 0.029). In the SP-pre group, no significant reduction in TH-positive midbrain neurons could be observed (Figure 3B, ipsi: 1459 ± 845.4 cells, contra: 1597 ± 1034 cells, *n* = 7, Wilcoxon matched pairs signed-rank test *p* = 0.297). Mesencephalic subfields showed no difference in TH-positive neuron counts either (VTA ipsi: 654.1 ± 445.7 cells, contra: 697.9 ± 502.6 cells, paired *t*-test *p* = 0.279; SNC ipsi: 663.4 ± 349.5 cells, contra: 729.0 ± 426.6 cells, paired *t*-test *p* = 0.258; RRF ipsi: 80.67 ± 37.34 cells, contra: 90.50 ± 30.02 cells, paired *t*-test *p* = 0.513). Likewise, total TH-positive cell count as well as analysis of mesencephalic subfields did not differ significantly between the ipsilesional and the contralesional side in SP-post animals (Figure 3C, ipsi: 1489 ± 479.1 cells, contra: 1760 ± 429.4 cells, *n* = 6, paired *t*-test *p* = 0.052; VTA: ipsi: 664.3 ± 219.5 cells, contra: 738.2 ± 189.4 cells, paired *t*-test *p* = 0.068; SNC ipsi: 637.2 ± 225.5 cells, contra: 796.0 ± 202.3 cells, paired *t*-test *p* = 0.074; RRF ipsi: 73.67 ± 37.59 cells, contra: 86.50 ± 36.54 cells, paired *t*-test *p* = 0.143). Between-group comparisons were performed by normalizing ipsi- to contralesional counts of TH-positive cells for each animal and mesencephalic subregion. The resulting ratio ipsilesional/contralesional (I/C ratio) indicated the extent of dopaminergic neurodegeneration. Here, a significant between-group difference emerged when all mesencephalic subregions were considered (I/C ratio control group: 0.69 ± 0.11, SP-pre: 0.93 ± 0.12, SP-post: 0.84 ± 0.17, 1-way ANOVA F(2, 16) = 0.22, *p* = 0.02). Post hoc analysis revealed a significant difference between the control and SP-pre group, whereas the SP-post group did not differ significantly from the other two (Figure 3D; Tukey’s multiple comparisons test control group vs. SP-pre: adjusted *p* = 0.016, control group vs. SP-post: adjusted *p* = 0.16, SP-pre vs. SP-post: adjusted *p* = 0.51). Analysis of mesencephalic subregions revealed a significant loss of TH-positive SNC neurons between SP-pre and the control group (I/C ratio control group: 0.61 ± 0.09, SP-pre: 0.92 ± 0.14, SP-post: 0.80 ± 0.22, 2-way ANOVA F(2, 16) = 1.68, *p* = 0.009; post hoc analyses between each of the groups with Tukey’s multiple comparisons test: control group vs. SP-pre: adjusted *p* = 0.007; control group vs. SP-post: adjusted *p* = 0.10, SP-pre vs. SP-post adjusted *p* = 0.41). Differences in I/C ratios in the other mesencephalic subregions failed to reach significance across groups (VTA: I/C ratio control group: 0.79 ± 0.15, SP-pre: 0.94 ± 0.13, SP-post: 0.89 ± 0.15, 1-way ANOVA F(2, 16) = 0.11, *p* = 0.19; RRF: I/C ratio control group: 0.70 ± 0.51, SP-pre: 0.94 ± 0.34, SP-post: 0.85 ± 0.20, 2-way ANOVA F(2, 14) = 0.62, *p* = 0.20).

### 2.3. Microglial Activation in Midbrain Was Not Increased by Substance P

**Figure 4:**
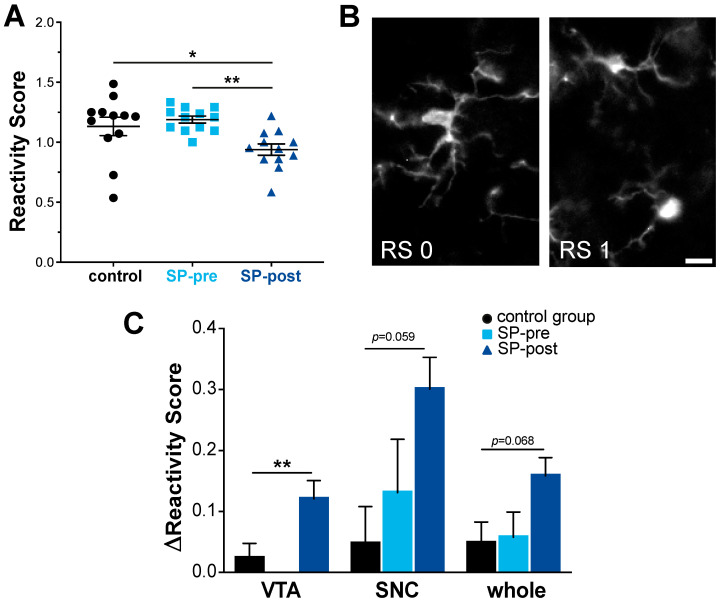
Microglial activation in the midbrain was not increased by Substance P. (**A**) Quantitative between-group comparison of overall ventral midbrain microglial reactivity scores (RS) after stroke (ipsilesional + contralesional hemisphere) indicates lowest values for SP-animals. (**B**) Representative microscopy image (10× magnification) illustrating different states of activation (RS 0 and RS 1) in Iba1-immunoreactive microglia. (**C**) Between-group assessments of ∆RS (RS ipsilesional – RS contralesional) indicate that a relevant ipsilesional increase in microglial activation was only present in SP-post animals. SNC: substantia nigra pars compacta, VTA: ventral tegmental area, whole: VTA + SNC, * *p* < 0.05, ** *p* < 0.01, data presented as mean ± SEM. Scale bar 10 μm.

Microglial activation was assessed based on morphology using the microglia reactivity score (RS, ranging from 0 to 4) for Iba1-positive midbrain cells (Figure 4B). Regarding the overall-RS (i.e., ipsi- and contralesional side), there was a significant effect of group (1-way ANOVA: F(2, 33) = 5.76, *p* = 0.0072; Figure 4A). Post hoc analysis revealed that reactivity scores were lowest in the SP-post group (Tukey’s multiple comparisons test control vs. SP-pre: adjusted *p* = 0.74, control group vs. SP-post: adjusted *p* = 0.045, SP-pre vs. SP-post: adjusted *p* = 0.0075), although microglia activation across all groups was not pronounced (RS CG: 1.13 ± 0.27, SP-pre: 1.19 ± 0.10, SP-post: 0.94 ± 0.16). For controls and SP-pre animals, there were no significant RS-differences between ipsi- and contralesional hemispheres (paired *t*-tests; CG: VTA: ipsi 0.63 ± 0.27, contra 0.61 ± 0.29, *p* = 0.36; SNC: ipsi: 1.1 ± 0.17, contra: 1.05 ± 0.15, *p* = 0.46, whole: ipsi 1.16 ± 0.25, contra 1.11 ± 0.30, *p* = 0.21; SP-pre: VTA: ipsi- and contra: 0.63 ± 0.21, SNC: ipsi 1.22 ± 0.24, contra 1.10 ± 0.20, *p* = 0.19, whole: ipsi 1.22 ±0.09, contra 1.16 ± 0.11, *p* = 0.22). For the SP-post group, a significantly higher microglial activation in the ipsilesional hemisphere was present across all subfields (paired *t*-tests; VTA: ipsi 0.82 ± 0.15, contra 0.70 ± 0.17, *p* = 0.009, SNC: ipsi 1.11 ± 0.10, contra 0.81 ± 0.15, *p* = 0.002, whole: ipsi 1.02 ± 0.12, contra 0.86 ± 0.17, *p* = 0.003). Between-group comparisons were made using the ∆RS (RS ipsilesional − RS contralesional) for each region. Here, significant differences were only present in the VTA (Figure 4C; ∆RS CG: 0.02 ± 0.06, SP-pre: 0.0 ± 0.0, SP-post: 0.12 ± 0.07, Kruskal–Wallis test *p* = 0.008). However, there were non-significant trends towards ipsilesional microglial activation in the SP-treated groups also for SNC and the whole dataset (SNC: ∆RS CG: 0.05 ± 0.15, SP-pre: 0.13 ± 0.21, SP-post: 0.30 ± 0.13, Kruskal–Wallis test *p* = 0.059; whole: ∆RS CG: 0.05 ± 0.08, SP-pre: 0.06 ± 0.1, SP-post: 0.16 ± 0.07, Kruskal–Wallis test *p* = 0.068). 

### 2.4. Substance P Prevents Hypertrophy of Endogenous SPergic Innervation after Stroke

**Figure 5:**
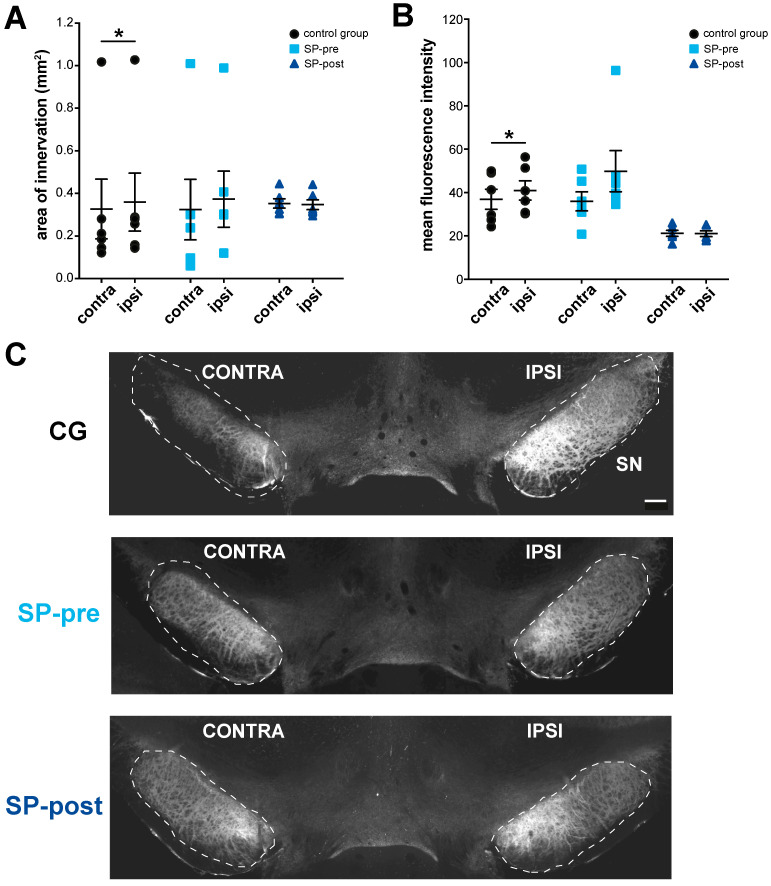
Substance P prevents ipsilesional hypertrophy of endogenous SPergic midbrain innervation after stroke. Comparisons of the area (**A**) and mean fluorescence intensity (**B**) of SP-immunoreactivity between hemispheres indicate an ipsilesional hypertrophy only in control animals. (**C**) Representative microscopy image (10× magnification) of SP-immunoreactivity in the ventral midbrain 15 days after stroke. Scale bar: 100 µm. Data presented with mean ± SEM, * *p* < 0.05. Scale bar: 100 μm.

Compared to the contralesional side, the control group exhibited significantly larger areas and higher mean fluorescence intensity values of SPergic innervation on the lesioned side (Figure 5A–C, area of innervation: ipsi 0.36 ± 0.33 mm^2^, contra 0.33 ± 0.34 mm^2^, Wilcoxon matched-pairs signed rank test *p* = 0.03; MFI: ipsi 40.95 ± 10.85, contra 36.92 ± 11.31, paired *t*-test *p* = 0.02, *n* = 6). For SP-pre animals, the ipsilesional area of SP-immunoreactivity was larger at trend level without differences for fluorescence intensities (area of innervation: ipsi 0.37 ± 0.32 mm^2^, contra 0.32 ± 0.35 mm^2^, Wilcoxon matched pairs signed-rank test *p* = 0.06; MFI: ipsi: 49.88 ± 23.29, contra: 35.95 ± 10.75, paired *t*-test *p* = 0.11, *n* = 6). No differences were present for the SP-post group (area of innervation: ipsi 0.35 ± 0.06 mm^2^, contra 0.35 ± 0.05 mm^2^, paired *t*-test *p* = 0.59, MFI: ipsi 21.03 ± 3.22, contra 21.17 ± 3.4, paired *t*-test *p* = 0.86, *n* = 6). 

## 3. Discussion

Intraperitoneal administration of SP facilitated motor rehabilitation in a rat model of motor cortical photothrombotic stroke. Here, injection immediately before training sessions (SP-pre) was more effective compared to the delayed administration (SP-post). SP furthermore prevented delayed dopaminergic cell loss after stroke, particularly in the SP-pre condition. Despite its proinflammatory propensity, SP administration did not cause an enlargement of stroke volumes or an activation of microglia within the midbrain. Finally, SP administration prevented an ipsilesional hypertrophy of striatal SPergic innervation, particularly within the SP-post condition. 

On first glance, preservation of dopaminergic neurons is the most likely explanation for the facilitatory effect of SP on motor rehabilitation as the outcome was best for the SP-pre group which also showed the least dopaminergic loss. In response to a photothrombotic stroke, ipsilesional dopaminergic cell death indicated by FJC-immunoreactivity occurred not later than seven days after ischemic lesion [9]. As loss of TH-positivity within the VTA was already present at this time point, dopaminergic degeneration in this compartment proceeded even faster. As a functional impairment can be expected to precede cell death and rehabilitation training started at day 3 after stroke, it is plausible that impaired dopaminergic signaling may have also affected the earlier phase of the rehabilitation training. However, with respect to learning curves (Figure 2), a substantial drug effect in SP-pre animals was already present between baseline training and the first rehabilitation training session (RT1), indicating an effect that occurred too early to be explained by protection of dopaminergic neurons. Interestingly, a similar early effect on motor learning was present in healthy rats that received intracortical SP injections within M1 30 min prior to a similar reaching training [29]. Thus, SP may facilitate motor learning as well as rehabilitation by promoting neuroplastic changes that are required for the storage of motor engrams: SP may facilitate the formation of novel circuits by increasing dendritic arborization and spine formation, as demonstrated in Purkinje cells of rats after intraventricular injections [34]. SP may further promote the formation of long-term plasticity as demonstrated in the visual cortex of rats [35] and the hippocampus of guinea pigs [36]. Finally, SP may increase the induction of c-Fos [37,38], an immediate early gene involved in motor learning [39] and motor rehabilitation [40]. In contrast to SP-pre, SP-post animals received the first injection 16 h after the first training session, a timing that should exclude the presence of relevant SP concentrations within the CNS during training [41]. Here, a rise in reaching performance can be merely observed within the second half of training (i.e., starting with RT 6), probably explained by the preservation of dopaminergic neurons that went into effect a week after ischemic stroke. 

Interestingly, protective effects in the SP-pre paradigm were more pronounced compared to SP-post group. For motor learning in healthy rats, dopaminergic neurons projecting to M1 are specifically activated during motor skill acquisition as indicated by c-Fos expression [42]. In cultured dopaminergic neurons, SP-mediated neuroprotection depends on activation of neurons, i.e., the sodium and calcium influx generated by excitatory synaptic inputs [25]. Thus, activation of dopaminergic neurons during rehabilitative training may create ideal conditions to exploit the protective propensity of SP in the SP-pre condition. In addition to its protective effect in cell culture, pretreatment with SP improved recovery and prevented dopamine loss in a model of subtotal nigrostriatal 6-hydroxydopamine lesion in rats [43]. The biological actions of SP are mainly mediated by the tachykinin NK1-receptor (NK1-R) that is highly expressed in the VTA and SN, but also in the cerebral cortex [44,45]. The NK1 receptor is a 7-transmembrane G-protein (Gq/G11) coupled receptor that mainly regulates the phosphoinositide pathway [22]. Via NK1-R activation and consecutive PKC and MAPK/ERK activation, SP protected cultured spinal ganglion neurons from trophic factor deprivation induced cell death by inhibiting caspase activation [24]. Moreover, SP prevented excitotoxic cell death in cultured cholinergic neurons evoked by the NMDA-agonist quinpirole [23]. Interestingly, blocking NMDA-receptors using MK-801 prevented dopaminergic degeneration after MCAO in mice [6]. Similarly, administration of the AMPA-antagonist YM872 after stroke prevented the atrophy of substantia nigra [46]. Thus, SP may exert protective effects due to anti-excitotoxic and anti-apoptotic mechanisms, whereas a certain degree of synaptic activation seems to support its neuroprotective propensity.

Apart from neuroprotection, SP is a potent mediator of neurogenic inflammation within the periphery [47] and central nervous system [33]. Post stroke, SP promotes vasodilation, microvascular permeability and edema formation and NK-1 receptor antagonism has been proposed as a neuroprotective approach to minimize post-ischemic injury [48,49,50]. Moreover, reduced SP levels in the area of secondary exo-focal degeneration after MCAO have been discussed to play a role in the inflammatory response within the ventral midbrain [31]. As the deteriorating inflammatory effects of SP are particularly relevant within the first 12 to 24 h after stroke [33,49], no significant differences in stroke volumes, mNSS values and motor deficits were observed across groups in this study. Furthermore, reactivity scores of microglia were overall low, arguing against a functionally relevant proinflammatory effect of SP in our model. In fact, overall reactivity scores were lowest in SP-post animals, although ipsilesional SP values were significantly increased compared to the contralesional side in this group. Thus, systemic administration of SP at day 3 post stroke is considerably safe.

As reported previously [9], ipsilesional endogenous SP-immunoreactivity was increased with respect to area and intensity in controls. With respect to the neuroprotective propensity of SP for dopaminergic neurons, it is tempting to speculate that this hypertrophy constitutes a compensatory mechanism in response to dopaminergic neurodegeneration. In line with this hypothesis, prevention of dopaminergic cell loss by systemic SP application also prevented the ipsilesional increment in SPergic innervation and preserved the physiological distribution of striato-nigral SP-containing synaptic terminals within the midbrain. 

## 4. Materials and Methods

### 4.1. Animals and Experiments

Adult 8–12-week-old male Sprague Dawley rats (*n* = 24; 280–320 g; Charles River, Sulzfeld, Germany) were used for this study. The skilled reaching task performed in this study is specifically validated for application in male rats, as female rats show steeper learning curves [51]. Biometric sample size assessments and power calculations were performed a priori in a biostatistical survey. Animals were housed in cages in groups of three with a 12/12 h light/dark cycle. Behavioral assessments were performed at the beginning of the light phase. Animals were food-deprived for 24 h prior to the first training session. Daily food intake was limited to ca. 50 g/kg body weight of standard chow, provided after each training session. Water was available ad libitum. Animal experiments were carried out in accordance with the ARRIVE guidelines, the EU Directive 2010/63/EU for animal experiments and were approved by the state of Baden-Württemberg under license number G-18/14. Chemicals were purchased from Sigma-Aldrich (Taufkirchen, Germany), unless noted otherwise. Experiments were conducted in line with the RIGOR criteria [52]: animals were randomly assigned to groups. Four animals had to be euthanized due to perioperative complications. Two animals completed behavioral assessments, but cardiac perfusion was unsuccessful. No further animal had to be excluded and all data is reported in the manuscript. With respect to surgery, behavioral assessments, tissue processing, staining procedures and histological analyses, researchers were blinded and not aware of group identities. 

### 4.2. Induction of Photothrombotic Motor Cortical Stroke

Induction of photothrombotic strokes was performed similar to a previous study [9]. In brief, rats were anesthetized with ketamine (75 mg/kg, i.p. Medistar, Ascheberg, Germany) and xylazine anesthesia (10 mg/kg, i.p. Bayer, Leverkusen, Germany). The head was fixed in a stereotaxic frame (Stoelting Co., Wood Dale, IL, USA). After a median skin incision, preparation and cleaning of the skull, a photothrombotic stroke was induced through the intact bone. In brief, Rose Bengal dye (10 mg/kg body weight; 7.5 mg/mL in sterile saline) was injected into the tail vein using a 24G venous line (Abbocath, Hospira, Maidenhead, UK) during the first 2 min of a 20 min illumination period using a cold light source (KL 1500, Schott AG, Mainz, Germany). To avoid calefaction damage, air-cooling was performed using a custom made ventilation system. To standardize lesion size, a 4 mm diameter stencil was placed above the forelimb area of the primary motor cortex (2 mm anterior and 2 mm lateral, relative to bregma). Blood oxygenation and heart rate were constantly monitored (MouseSTAT Pulse Oximeter for mice and rats, Kent Scientific Corporation, Torrington, CT, USA), body temperature was controlled using a heating pad (Temperature Controller TC-1000, CWE Inc., Ardmore, PA, USA). Carprofen (5 mg/kg, s.c.; Norbrook, Newry, Northern Ireland) was given after surgery for pain relief.

### 4.3. Behavioral Paradigm, Drug Application and Assessment of Post-Stroke Deficits

The experimental protocol is summarized in Figure 1A. Reaching training was performed similar to Whishaw and Pellis, 1990 [53]. Rats were trained to reach and grasp for a food pellet (45 mg, Bio-serve, Frenchtown, NJ, USA)., placed on a ledge outside of a training cage (15 × 25 × 25 cm) with a vertical window (1 cm wide, 5 cm high). As pre-training, food pellets were placed on the ledge at a distance of 15 mm in front of the window. In this position, pellets were only retrievable by forelimb reaching. The paw that was used most frequently was defined as the preferred side. Reaching training started after animals performed 50 reaching attempts (trials) in less than 20 min on two consecutive pre-training days. Reaching training was initiated by shifting the pellet to align with the edge of the window, allowing the use of the preferred limb only. Furthermore, a metal bar (2 mm diameter) was placed as a sill in front of the window so that animals had to reach over this fence to grasp the pellet. Each reaching trial was scored as “successful” (reach, grasp and retrieve) or “unsuccessful”. Each training session consisted of 48 trials or 30 min, whichever came first. Reaching performance was defined as the number of successful trials out of 48 possible trials (success rate) in percent. The duration of training sessions was assessed as a measure of attention and motivation. Rats completed five training sessions ensuring that they had reached a performance plateau before the stroke (T1-5, Figure 1A). The average success rate of the last two sessions (T4-5) was used as a measure of plateau performance. Starting 2 days after stroke, animals were re-trained for 24 trials (i.e., “base” or B; Figure 1A) to assess the post-stroke deficit. Then, animals underwent re-training for ten sessions on consecutive days (RT1-10; Figure 1A). The “base” was subtracted from the average success rate of the last 3 re-training sessions (RT8-10) to compute rehabilitation plateaus. Before the “base” assessment, animals were randomly assigned to three groups by drawing lots: a control group receiving 3 mL/kg [54] saline i.p. 30 min prior to training (CG, *n* = 7); a group receiving substance P 250 µg/kg i.p. in a volume of 3 mL/kg saline 30 min prior to training (SP-pre; *n* = 7); a group receiving substance P 250 µg/kg i.p. in a volume of 3 mL/kg saline 16 h after training (SP-post; *n* = 6). In the SP-pre group, systemic administration of SP should exert its effects during training [26] similar to a previous study [29]. As CNS-effects of systemically administered SP decay after a few hours [41], timing of injection for SP-post animals (i.e., 16 h post- and 8 h pre-training) allow the assessment of SP-effects independently from training. General post-stroke deficits were assessed using the Modified neurological severity score (mNSS) one day before and after stroke induction and immediately before rats were perfused (P; Figure 1A). The mNSS is a widely used and valid tool to evaluate neurological functional deficits in rodents after unilateral brain injury [55]. Neurological function is based on motor, sensory, reflex, and balance tests and graded on a scale of 0 to 18 (normal score 0; maximal deficit score 18). As the absence of a neurological deficit (mNSS = 0) would question the presence of a relevant ischemic lesion, a mNSS of 0 was an exclusion criterion for our study. However, no animal had to be excluded due to this reason. Training, re-training and mNSS-assessment were documented by video-footage (HERO3+, GoPro, San Mateo, CA, USA) and analyzed off-line. The researcher performing training and video-analysis was blinded with respect to group identities (i.e., substance P vs. saline; injection pre- vs. post-training). 

### 4.4. Euthanasia and Tissue Processing

Animals were deeply sedated (ketamine 80 mg/kg i.p., Medistar, Ascheberg, Germany; xylazin 12 mg/kg i.p., Bayer, Leverkusen, Germany) and perfused transcardially with 4% paraformaldehyde (PFA). Brains were quickly removed and kept in 4% PFA for 24 h before being transferred to 30% sucrose solution. Coronal sections were prepared using a sliding microtome with freezing stage (Leica Microsystems GmbH, Wetzlar, Germany). For analysis of the stroke placement, brain slices of 30 μm thickness were prepared +4.0 to −2.5 mm with respect to bregma and alternately collected in seven sampling wells. Thus, a sample contains every 7th section with a fixed distance of 210 μm between subsequent sections. For the analysis of dopaminergic midbrain nuclei, brain slices of 40 μm thickness were prepared −5 to −7 mm with respect to bregma and alternately collected in five sampling wells. Thus, a sample contains every 5th section with a fixed distance of 200 μm between subsequent sections. All coordinates are based on Paxinos and Watson, 2014 [56].

### 4.5. Histochemistry and Immunohistochemistry

*FluoroJade-C (FJ-C) staining:* brain sections were washed in 0.05 M tris-buffered saline (TBS) for 5 min, mounted on glass slides coated with 0.3% gelatine and then were dried for 2 h in an incubator at 50 °C. Slides were rinsed in 1% sodium hydroxide diluted in 80% ethanol for 5 min. They were then dehydrated in a graded series (ethanol 70% for 2 min, 0.45 M natrium chloride solution for 2 min) and subsequently incubated in 0.006% potassium permanganate for 10 min. The slides were washed with distilled water for 1 min and then placed in 0.0001% FluoroJade C solution (Chemicon, Etobicoke, Canada) for 10 min. Lastly, the slides were washed three times in distilled water for 1 min, dried in an incubator at 40 °C for 1 h and cleared in xylol for 1 min. Then, slides became cover slipped with Depex mounting medium (Electron Microscopy Sciences, Hatfield, PA, USA).

*Immunohistochemistry:* free floating sections were rinsed three times in 0.05 M TBS, treated with 3% H_2_O_2_ for 30 min, washed three times in 0.05 M TBS, then rinsed in 0.1% Triton for 10 min, and blocked for 30 min in 10% fetal cow serum. Sections were incubated with primary antibody diluted in 0.05 M TBS and 5% fetal cow serum for 24 h at 4 °C under agitation. The following primary antibodies were used: 1:400 monoclonal mouse anti-tyrosine hydroxylase antibody (anti-TH, Chemicon International, Temecula, CA, USA, cat. MAB 318); 1:1000 polyclonal rabbit anti-ionized calcium-binding adapter molecule 1 antibody (anti-Iba1, Wako, Neuss, Germany, cat. 019-19741); 1:500 monoclonal mouse anti-Substance P antibody (anti-SP, R&D Systems, Abingdon, UK, cat. MAB 4375). Sections were then washed three times in 0.05 M TBS and subsequently incubated with corresponding secondary antibodies: goat anti-rabbit fluorescein isothiocyanate (FITC)-coupled (Thermo Fisher Scientific, Darmstadt, Germany, cat. F2765) or goat anti-mouse Cyanine3 (Cy3)-coupled (Thermo Fisher Scientific, Darmstadt, Germany, cat. A10521) diluted 1:200 in 0.05 M TBS and 2.5% fetal cow serum at 4 °C for 90 min. Sections were mounted with Vectashield (Vector Laboratories Inc., Burlingame, CA, USA). 

### 4.6. Histological Analysis

Images of brain sections were digitized using a fluorescent microscope (Axioplan II, Zeiss AG, Jena, Germany; equipped with a motorized x–y stage; 10×/0.5 EC Plan-Neofluar objective) and analyzed using Fiji software [57]. Every 7th section was used for assessment of lesion placement and volume. Lesioned tissue was identified by FJ-C fluorescence signal indicating degenerating cells (Figure 1B). Lesion volume was computed using the frustum-formula V = h × π3⋅((r1)2 + r1⋅r2 + (r2)2) based on the measured lesion area and the distance between subsequent sections (210 μm). Extensions of ischemic lesions were compared to the Paxinos and Watson [56] atlas to ensure congruence with motor cortical topography. Even though the largest part of the lesion was confined to M1, tails invading the secondary motor cortex (M2) existed at the rostral pole of the stroke. Every photothrombotic lesion was restricted to the cortex and there was no violation of the corpus callosum or striatum. No animal had to be excluded due to misplacement of photothrombotic stroke. Dopaminergic midbrain nuclei were identified based on TH positivity, as TH was confirmed to be the most reliable marker for dopaminergic neurons in the rodents’ midbrain [58]. The nomenclature of dopaminergic structures was adopted from Dahlström and Fuxe [59]: nucleus A8 contains the retrorubral field (RRF), nucleus A9 contains the substantia nigra pars compacta (SNC). Nucleus A10 contains the ventral tegmental area (VTA: including nucleus paranigralis, parabrachial pigmented nucleus and rostral linear nucleus raphe), the central linear nucleus (CLi) and the interfascicular nucleus (IF). For quantification of anti-TH and anti-Iba1 positive cells, every 5th section was taken into account. A total of six to eight midbrain slices were analyzed per animal. Positive cells were counted in the entire section using the cell counter plugin of Fiji. For the quantification of anti-SP signal in midbrain sections, every 5th section was processed. The mean area of innervation and mean signal intensities were assessed applying the measure routine of Fiji. Activation of microglia (anti-Iba1 positive) was qualitatively assessed by cell morphology and graded from 0 (no reactivity) to 4 (severe reactivity) [9,60]. This reactivity score (RS) was assessed independently for mesencephalic subfields and in each section assigned to quantification to obtain average values for each region.

### 4.7. Statistical Analyses

Statistical analyses and graph presentations were performed using Prism version 8 (GraphPad Software, La Jolla, CA, USA) and SPSS version 25 (IBM, Ehningen, Germany). For all tests, normal distribution was checked using the Shapiro–Wilk test for normality. For ANOVAs, equality of variances was confirmed using the Brown–Forsythe test. Animals were designated as subjects for the analysis. Paired *t*-tests were used for within-group comparisons (e.g., lesioned vs. non-lesioned hemisphere). For between group comparisons, 1-way ANOVAs or the nonparametric Kruskal–Wallis-test were used. Post hoc analyses to assess individual differences between two of the three examined groups were performed with Tukey’s multiple comparisons test. For analysis of anti-TH and anti-SP signals, an ipsi/contra ratio was calculated. For analysis of reactivity scores (RS), the difference Δ between ipsi- and contralesional hemispheres was calculated. Learning curves and evolution of mNSS values were compared using an RM-ANOVA, with factors group and time. The sphericity assumption was tested using the Mauchly criterion and the Greenhouse–Geisser correction was used, where appropriate. For training and retraining curves, performance during the first training session (training) or the “base” (re-training) was added as a covariate to avoid false-positive results caused by baseline differences. Numerical results were expressed as mean and standard deviation (SD). 

## Data Availability

The data that support the findings of this study are available from the corresponding author upon reasonable request.
